# Supplementary material for: Internalizing and externalizing mental health problems affect in-school adolescent’s health-related quality of life in eastern Ethiopia: A cross-sectional study
Source: PLoS One. 2022 Aug 4;17(8):e0272651. doi: 10.1371/journal.pone.0272651 (PMC9352091; doi:10.1371/journal.pone.0272651)
Supplement: S2 Table — (DOCX) [file pone.0272651.s002.docx]

**S2 Table. Ordinal Logistic Regression analyses Showing the association between environmental related variables, mental health problems and HrQoL among In-School Adolescents in Harari Region, Eastern Ethiopia, 2020, (n = 3227).**

| **Variables** | **HrQoL of adolescents** | | | **COR (95% CI)** | **AOR (95% CI)** |
| --- | --- | --- | --- | --- | --- |
|  | **Low (%)** | **Medium (%)** | **High (%)** |  |  |
| **Residence** (Ref Urban) | 19.40 | 52.20 | 28.50 | 1.0 | 1.0 |
| Rural | 40.90 | 49.50 | 9.60 | 0.30 (0.10, 0.40)* | 0,50 (0.40, 0.60)* |
| **School type** (Ref Private) | 25.60 | 51.80 | 22.60 | 1.0 | 1.0 |
| Public | 17.20 | 51.60 | 31.20 | 1.60 (1.40, 1.80)* | 0.90 (0.70, 0.90)* |
| **Wealth index** (Ref Lowest) | 30.50 | 50.70 | 18.80 | 1.0 | 1.0 |
| Middle | 17.10 | 53.80 | 29.0 | 1.90 (1.70, 2.20)* | 1.40 (1.20, 1.70)* |
| Highest | 18.90 | 49.80 | 31.40 | 2.00 (1.60, 2.40)* | 1.50 (1.20, 1.80)* |
| **Internalizing problem** (Ref Normal) | 17.70 | 53.00 | 29.30 | 1.0 | 1.0 |
| Borderline | 36.20 | 45.30 | 18.60 | 0.40 (0.30, 0.60)* | 0.60 (0.90, 0.70)* |
| Abnormal | 40.10 | 48.80 | 11.10 | 0.30 (0.30, 0.40)* | 0.45 (0.40, 0.60)* |
| **Externalizing problems** (Ref Normal) | 20.20 | 52.30 | 27.50 | 1.0 | 1.0 |
| Borderline | 38.00 | 48.80 | 13.20 | 0.40 (0.30, 0.60)* | 0.61 (0.40, 0.90)* |
| Abnormal | 43.90 | 47.70 | 8.30 | 0.30 (0.20, 0.40)* | 0.50 (0.40, 0.70)* |

Note. HrQoL: health-related quality of life, CI: confidence interval, COR: crude odds ratio, AOR: adjusted odds ratio, *statistically significant with a p-value of less than 0.05, 1.0: indicates reference categories
