# Supplementary material for: Internalizing and externalizing mental health problems affect in-school adolescent’s health-related quality of life in eastern Ethiopia: A cross-sectional study
Source: PLoS One. 2022 Aug 4;17(8):e0272651. doi: 10.1371/journal.pone.0272651 (PMC9352091; doi:10.1371/journal.pone.0272651)
Supplement: S3 Table — (DOCX) [file pone.0272651.s003.docx]

**S3 Table. Ordinal Logistic Regression analyses Showing the association between family related variables, mental health problems and HrQoL among In-School Adolescents in Harari Region, Eastern Ethiopia, 2020, (n = 3227).**

| **Variables** | **HrQoL of adolescents** | | | | | **COR (95% CI)** | **AOR (95% CI)** |
| --- | --- | --- | --- | --- | --- | --- | --- |
|  | **Low (%)** | **Medium (%)** | | **High (%)** | |  |  |
| **Parental marital status** (Ref Living together) | 22.20 | | 51.00 | | 26.90 | 1.0 | 1.0 |
| Living Separated | 26.30 | | 55.00 | | 18.80 | 0.70 (0.60, 0.90)* | 0.70 (0.50, 0.90)* |
| Divorced or widowed | 24.30 | | 53.90 | | 21.70 | 0.80 (0.70, 0.90)* | 0.80 (0.64, 0.90)* |
| **Family size** (Ref ≤ 3) | 17.90 | | 54.60 | | 27.50 | 1.0 | 1.0 |
| 4 to 7 | 21.30 | | 51.40 | | 27.30 | 0.90 (0.70 , 1.10) | 0.93(0.73, 1.11) |
| ≥ 8 | 30.90 | | 51.30 | | 17.80 | 0.50 (0.40, 0.60)* | 0.60 (0.50, 0.70)* |
| **History of parental mental illness** (Ref No) | 20.40 | | 52.20 | | 27.40 | 1.0 | 1.0 |
| Yes | 39.50 | | 48.40 | | 12.10 | 0.40 (0.30, 0.50)* | 0.50 (0.40, 0.60)* |
| **Internalizing problem** (Ref Normal) | 17.70 | | 53.00 | | 29.30 | 1.0 | 1.0 |
| Borderline | 36.20 | | 45.30 | | 18.60 | 0.40 (0.30, 0.60)* | 0.50 (0.40, 0.70)* |
| Abnormal | 40.10 | | 48.80 | | 11.10 | 0.30 (0.30, 0.40)* | 0.40 (0.30, 0.50)* |
| **Externalizing problems** (Ref Normal) | 20.20 | | 52.30 | | 27.50 | 1.0 | 1.0 |
| Borderline | 38.00 | | 48.80 | | 13.20 | 0.40 (0.30, 0.60)* | 0.6 (0.4, 0.8)* |
| Abnormal | 43.90 | | 47.70 | | 8.30 | 0.30 (0.20, 0.40)* | 0.50 (0.40, .70)* |

Note. HrQoL: health-related quality of life, CI: confidence interval, COR: crude odds ratio, AOR: adjusted odds ratio, *statistically significant with a p-value of less than 0.05, 1.0: indicates reference categories
