# Supplementary material for: Internalizing and externalizing mental health problems affect in-school adolescent’s health-related quality of life in eastern Ethiopia: A cross-sectional study
Source: PLoS One. 2022 Aug 4;17(8):e0272651. doi: 10.1371/journal.pone.0272651 (PMC9352091; doi:10.1371/journal.pone.0272651)
Supplement: S4 Table — (DOCX) [file pone.0272651.s004.docx]

**S4 Table. Analyses based on Ordinal Logistic Regression Family-related characteristics, mental health concerns, and HrQoL among In-School Adolescents in Harari Region, Eastern Ethiopia, 2020 (n = 3227).**

| **Variables** | **HrQoL of adolescents** | | | **COR (95% CI)** | **AOR (95% CI)** |
| --- | --- | --- | --- | --- | --- |
|  | **Low (%)** | **Medium (%)** | **High (%)** |  |  |
| **Age** (Ref 13 to 15) | 20.40 | 49.40 | 30.20 | 1.0 | 1.0 |
| 16 to 19 | 25.00 | 53.90 | 21.10 | 0.70 (0.60, 0.80)* | 0.70 (0.60, 0.80)* |
| **Sex** ( Ref Male) | 25.40 | 50.80 | 23.80 | 1.0 | 1.0 |
| Female | 20.40 | 52.60 | 27.00 | 1.30 (1.10, 1.40)* | 1.10 (0.92, 1.20) |
| **Alcohol use** (Ref Never use) | 21.40 | 51.90 | 26.70 | 1.0 | 1.0 |
| Ever use | 34.40 | 50.40 | 15.20 | 0.50 (0.40, 0.60)* | 0.80 (0.60, 0.90)* |
| **Tobacco use** (Ref Never use) | 21.50 | 52.20 | 26.30 | 1.0 | 1.0 |
| Ever use | 45.40 | 43.90 | 10.80 | 0.30 (0.20, 0.40)* | 0.80 (0.57, 1.20) |
| ***Khat use*** *(Ref* Never use*)* | 21.20 | 51.80 | 26.90 | 1.0 | 1.0 |
| Ever use | 31.30 | 51.30 | 17.50 | 0.60 (0.50, 0.70)* | 0.90 (0.80, 1.20) |
| **Any chronic medical conditions** (Ref No) | 20.90 | 51.50 | 27.70 | 1.0 | 1.0 |
| Yes | 30.20 | 52.80 | 17.00 | 0.60 (0.50, 0.70)* | 0.80 (0.70, 0.90)* |
| **Residence** (Ref Urban) | 19.40 | 52.20 | 28.50 | 1.0 | 1.0 |
| Rural | 40.90 | 49.50 | 9.60 | 0.30 (0.10, 0.40)* | 0.50 (0.40, 0.70)* |
| **School type** (Ref Private) | 25.60 | 51.80 | 22.60 | 1.0 | 1.0 |
| Public | 17.20 | 51.60 | 31.20 | 1.60 (1.40, 1.80)* | 0.90 (0.77, 1.10)* |
| **Wealth index** (Ref Lowest) | 30.50 | 50.70 | 18.80 | 1.0 | 1.0 |
| Middle | 17.10 | 53.80 | 29.00 | 1.90 (1.70, 2.20)* | 1.40 (1.20, 1.60)* |
| Highest | 18.90 | 49.80 | 31.40 | 2.00 (1.60, 2.40)* | 1.40 (1.20, 1.70)* |
| **Parental marital status** (Ref Living together) | 22.20 | 51.00 | 26.90 | 1.0 | 1.0 |
| Living Separated | 26.30 | 55.00 | 18.80 | 0.70 (0.60 to 0.90)* | 0.70 (0.50, 0.80)* |
| Divorced or widowed | 24.30 | 53.90 | 21.70 | 0.80 (0.70, 0.90)* | 0.90 (0.60, 0.90)* |
| **Family size** (Ref ≤ 3) | 17.90 | 54.60 | 27.50 | 1.0 | 1.0 |
| 4 to 7 | 21.30 | 51.40 | 27.30 | 0.90 (0.70, 1.10) | 0.90 (0.70, 10.10) |
| ≥ 8 | 30.90 | 51.30 | 17.80 | 0.50 (0.40, 0.60)* | 0.77 (0.60, 0.90)* |
| **History of mental illness in the family** (Ref No) | 20.40 | 52.20 | 27.40 | 1.0 | 1.0 |
| Yes | 39.50 | 48.40 | 12.10 | 0.40 (0.30, 0.50)* | 0.60 (0.50, 0.80)* |
| **Internalizing problem** (Ref Normal) | 17.70 | 53.00 | 29.30 | 1.0 | 1.0 |
| Borderline | 36.20 | 45.30 | 18.60 | 0.40 (0.30, 0.60)* | 0.60 (0.45, 0.80)* |
| Abnormal | 40.10 | 48.80 | 11.10 | 0.30 (0.30, 0.40)* | 0.50 (0.40, 0.60)* |
| **Externalizing problems** (Ref Normal) | 20.20 | 52.30 | 27.50 | 1.0 | 1.0 |
| Borderline | 38.00 | 48.80 | 13.20 | 0.40 (0.30, 0.60)* | 0.60 (0.50, 0.90)* |
| Abnormal | 43.90 | 47.70 | 8.30 | 0.30 (0.20, 0.40)* | 0.60 (0.50, 0.80)* |

Note. HrQoL: health-related quality of life, CI: confidence interval, COR: crude odds ratio, AOR: adjusted odds ratio, *statistically significant with a p-value of less than 0.05, 1.0: reference categories
