# Supplementary material for: Internalizing and externalizing mental health problems affect in-school adolescent’s health-related quality of life in eastern Ethiopia: A cross-sectional study
Source: PLoS One. 2022 Aug 4;17(8):e0272651. doi: 10.1371/journal.pone.0272651 (PMC9352091; doi:10.1371/journal.pone.0272651)
Supplement: Attachment — Submitted filename: Review.docx [file pone.0272651.s006.docx]

**High scores of both internalizing and externalizing mental health problems were the major factors affecting in-school adolescent’s health-related quality of life in eastern Ethiopia: A cross-sectional study**

The authors describe a cross-sectional study with the objective to examine the associations between mental health problems and health-related quality of life among school adolescents aged 13–19 years, in the Harari region, eastern Ethiopia.

Topic of interest to readers. The manuscript it is easy to follow, is well written and provides an important contribution to the field and the conclusions agree with the data presented.

Discussion is clear and nicely places study findings in context with other studies. The findings are certainly of interest to the readers of Plos One.

Nevertheless, I have some minor issues below for authors to consider.

When an abbreviation is used for the first time, you should use it throughout all the text. This happens with health-related quality of life (HrQoL).

Abstract - Please add the sample size.

Methods - Please provide the Cronbach's alpha results for reliability and validity.

Line 330 – a reference is need to support the sentence.
